# Abnormal Chromatin Clumping in Myeloblasts Mimicking Chronic Lymphocytic Leukaemia: A Diagnostic Pitfall

**DOI:** 10.7759/cureus.20658

**Published:** 2021-12-24

**Authors:** Tushar Sehgal, Malvika Gaur, Ganesh Kumar, Arulselvi Subramanian

**Affiliations:** 1 Laboratory Medicine, All India Institute of Medical Sciences, New Delhi, New Delhi, IND; 2 Hematology, All India Institute of Medical Sciences, New Delhi, New Delhi, IND

**Keywords:** chronic lymphocytic leukaemia, hematological neoplasm, flow cytometry, myeloblasts, abnormal chromatin clumping

## Abstract

Abnormal chromatin clumping (ACC) in cells of myeloid lineage is a distinct morphological entity. It has been described mainly in polymorphs in haematological neoplasms involving myelodysplasia or myeloproliferation. We here describe a rare case of ACC in myeloblasts in an elderly man that mimicked chronic lymphocytic leukaemia. Flow cytometry played a crucial role in characterizing the myeloid lineage of the blasts, thus avoiding a misdiagnosis. To the best of our knowledge, this is the third time such a case has been reported in the literature.

## Introduction

Abnormal chromatin clumping (ACC) first described by Gustke et al. is a morphological peculiarity of myeloid cells seen in haematopoietic neoplasms [1,2]. It is a nuclear abnormality seen especially in polymorphonuclear cells in both peripheral blood and bone marrow as an exaggerated form of chromatin clumping [1]. A few authors debate it as a clue to a new subtype of myelodysplastic syndrome (MDS) while others categorize it as an entity with both myelodysplastic and myeloproliferative features [2,3]. The WHO book on the classification of hematopoietic and lymphoid neoplasm also describes ACC as a morphological feature associated with both myeloproliferation and MDS [4]. We describe an unusual and rare case of ACC in myeloblasts in an elderly man with acute myeloid leukaemia (AML) that mimicked chronic lymphocytic leukaemia (CLL). Recognizing this atypical morphology is important as both neoplasms (AML and CLL) differ in their treatment and prognosis.

## Case presentation

**Figure 1:**
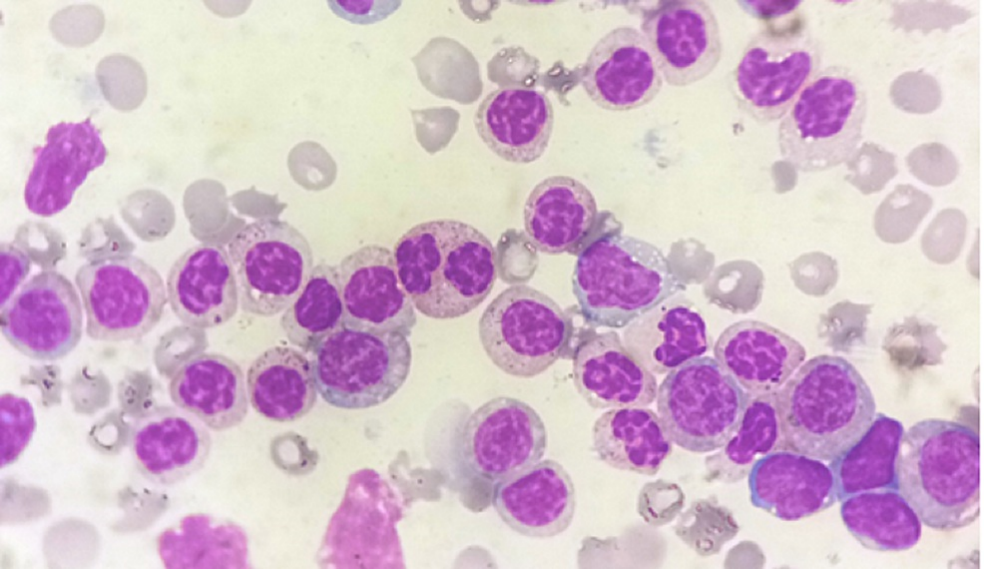
PBF revealed atypical mononuclear cells with abnormal nuclear chromatin (dark areas of chromatin separated by clear zones) mimicking the “soccer ball” chromatin of CLL. PBF - Peripheral blood film, CLL - chronic lymphocytic leukaemia

A 95-year-old man presented to the emergency department with fatigue and dyspnoea for two to three weeks. Complete blood count showed haemoglobin of 63 g/L, platelet count of 27 × 10^9^/L and white blood cell count of 177 × 10^9^/L. He had mild hepatosplenomegaly and no lymphadenopathy. Biochemical parameters showed CRP of 22.7 mg/L (normal <3 mg/L), blood urea of 82 mg/dL (normal 5-20 mg/dL), uric acid of 9.7 mg/dL (normal 3.4-7 mg/dL) and serum ferritin of 1,057 ng/mL (normal 20-250 ng/mL). Peripheral blood film (PBF) revealed 80% atypical mononuclear cells with abnormal nuclear chromatin (dark areas of chromatin separated by clear zones) and inconspicuous nucleoli mimicking a CLL (Figure 1). No Auer rods were seen. Granulocytes or any other leukocyte did not show ACC.

**Figure 2:**
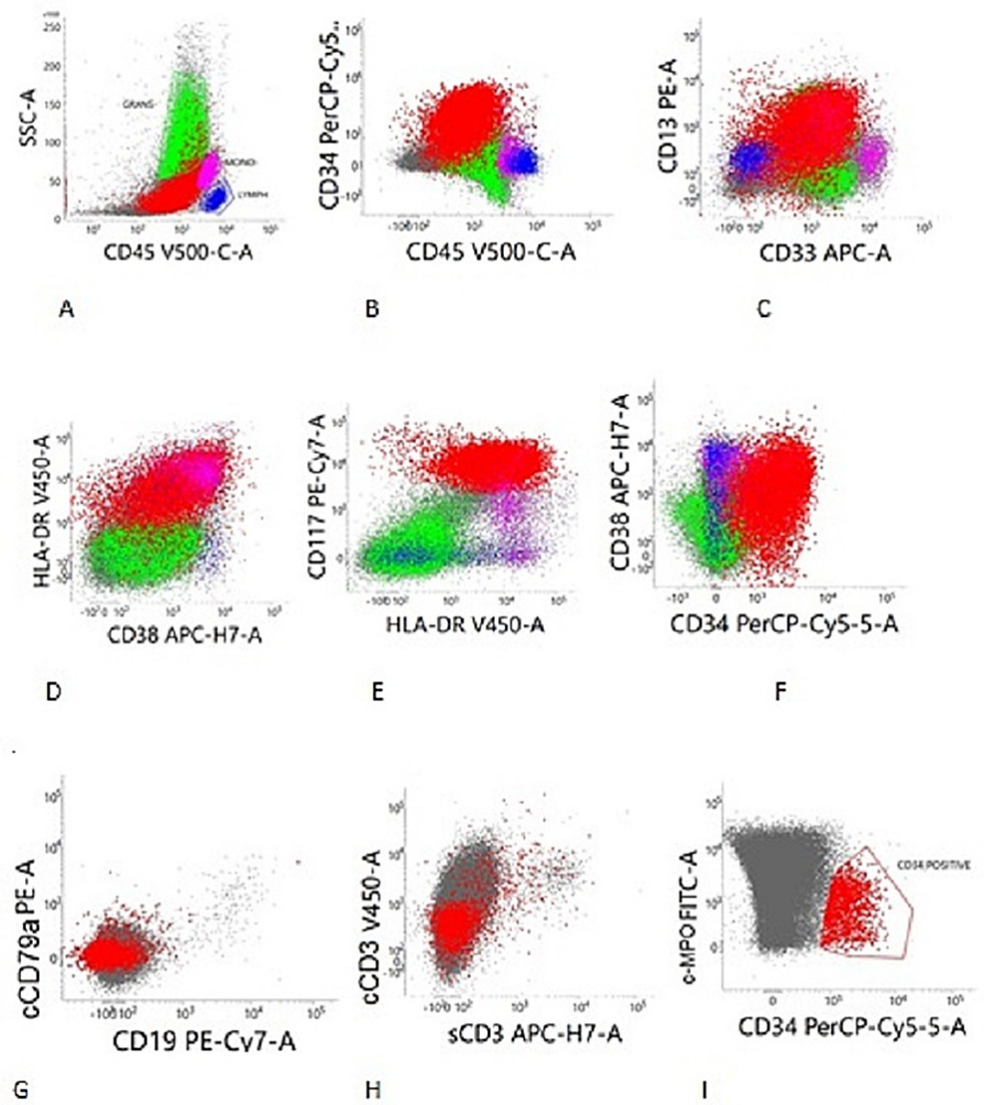
(A-I) Flow cytometry immunophenotyping using the CD45 vs SSC gating strategy revealed an abnormal cell population (blasts) in the dim to moderate CD45 region. The blast population was positive for CD13, CD33, CD117, CD34, CD38 and HLA-DR. The acute leukaemia orientation tube (ALOT) showed the blast population that was positive for cMPO and negative for CD19, cCD79a, sCD3 and cCD3.

The white cell differential fluorescence (WDF) scatterplot obtained by the Sysmex XN series haematology analyzer showed an abnormal cell population in the atypical lymphoid/blast region. Immunophenotyping by flow cytometry revealed a dim to moderate CD45 positive population comprising 70% of all singlets with a low to medium side scatter (SSC) in the CD45 vs SSC plot. The blasts were positive for CD13, CD33, CD117, CD34, cMPO, HLA-DR and CD38 and were negative for cCD3, CD10, CD19 and CD20 thus confirming the diagnosis of AML (Figures 2A-2I). He was started on azacytidine-based therapy, broad-spectrum antibiotics and transfusion support. Genetic analysis could not be performed as the patient succumbed to his illness on the second day of admission due to bilateral pneumonia, sepsis and multiorgan dysfunction syndrome due to AML.

## Discussion

ACC in leucocytes is characterized by an abnormal clumping of heterochromatin into large blocks that is separated by clear zones of euchromatin and that mimic nuclear fragmentation. Ultrastructurally, the heterochromatin gets distributed in the form of peripheral blocks, which may cause the nuclear membrane to bulge out without getting disrupted. The granules and other cytoplasmic structures appear normal [2].

The 2016 update of the WHO textbook of hematopoietic and lymphoid neoplasm describes dysgranulopoiesis as an important criterion for the diagnosis of dysplasia in both MDS and AML. Some of the features of dysgranulopoiesis include the small or unusually large size of polymorphs, nuclear hyposegmentation or hypersegmentation, decreased granules or agranularity of the cytoplasm, pseudo-Chediak-Higashi granules, Dohle bodies and Auer rods [4]. ACC is mentioned in the context of dysgranulopoiesis in atypical chronic myeloid leukaemia (aCML), BCR-ABL 1-negative and primary myelofibrosis [4]. Most cases reported as the syndrome of ACC can be considered a variant of aCML. These cases are characterized by an increased percentage of neutrophils and precursors in the blood and bone marrow that show an unusual clumping of the nuclear chromatin [4,5]. ACC in granulocytes has also been observed in five renal transplant patients (three young adults and two children) and one paediatric case of bone marrow transplantation who was treated with immunosuppressive drug therapy particularly mycophenolate mofetil. The mechanism of the appearance of ACC is debated although the direct toxic effect of mycophenolate mofetil is one of the hypotheses. In five patients ACC disappeared after tapering or discontinuation of mycophenolate mofetil, confirming that ACC was transient and dose-dependent [6]. ACC has also been described due to an alteration of the immune status. Acquired ACC in granulocytes has been observed in human immunodeficiency virus patients and some patients treated for lymphoproliferative disorders [6,7]. ACC in myeloblasts has been reported in association with AML on two previous occasions (Table 1) [8,9].

**Table 1:** Review of literature on ACC in myeloblasts in AML List of abbreviations: Hb.: haemoglobin; WBC: white blood cells; Plt.: platelets; ACC: abnormal chromatin clumping; AML: acute myeloid leukaemia

Study	Age/gender	CBC	Flow cytometry	Diagnosis	Cytogenetics
Skeith et al. [8] 2016	73/M	Hb. 81 g/L, WBC 12.4 × 10^9^/L, Plt 28 × 10^9^/L	CD13, CD33, CD34, CD117, HLA-DR, CD7 and CD11b	AML	Monosomy 20 and Inversion 7
Shang et al. [9] 2020	49/M	Hb. 73 g/L, WBC 110 × 10^9^/L, Plt 636 × 10^9^/L, Basophils-12.4 × 10^9^/L	CD13, CD33, CD34, CD117, HLA-DR, CD36 and MPO	AML with basophilia	46,XY,t(4;12)(q12;p13)
Current study 2021	95/M	Hb. 63g/L, WBC 177 × 10^9^/L, Platelet 27 × 10^9^/L	CD13, CD33, CD34, CD117, HLA-DR, CD38 and MPO	AML	-

In our case, the patient was an elderly individual who presented with fatigue and dyspnoea. PBF showed atypical mononuclear cells that morphologically appeared like lymphoid cells of CLL. CLL is a mature and indolent neoplasm that presents with an increased percentage of small mature lymphocytes, along with the presence of smudge cells. CLL lymphocytes are often referred to as “soccer-ball” lymphocytes because the nucleus is mature, dark, and can have a “cracked” appearance [10]. The atypical morphology due to abnormal nuclear clumping is a diagnostic pitfall and led to initial suspicion of CLL. However, immunophenotyping studies by flow cytometry played a vital role in establishing the myeloid lineage of the atypical cells (blasts) thus avoiding an incorrect diagnosis.

## Conclusions

This case describes a rare and unusual morphology of blasts in AML. Due to the paucity of these cases in the literature, it would be currently difficult to assess the impact of this abnormal morphology on prognosis in patients with AML. More cases need to be studied and analyzed to draw an appropriate conclusion. The hematopathologists and laboratory physicians must be mindful of this infrequent occurrence in AML and perform immunophenotyping by flow cytometry for a prompt diagnosis and accurate treatment.
